# Tyrosine Kinase Receptors Axl and MerTK Mediate the Beneficial Effect of Electroacupuncture in a Cuprizone-Induced Demyelinating Model

**DOI:** 10.1155/2020/3205176

**Published:** 2020-07-04

**Authors:** Zaofeng Zou, Jingxian Sun, Zheng Kang, Yumeng Wang, Hui Zhao, Keying Zhu, Jun Wang

**Affiliations:** ^1^Department of Integrative Medicine and Neurobiology, School of Basic Medical Sciences, Shanghai Medical College, Fudan University, Shanghai, China; ^2^State Key Laboratory of Medical Neurobiology and MOE Frontiers Center for Brain Science, Institute of Brain Science, Fudan University, Shanghai, China; ^3^Department of Integrative Medicine, Huashan Hospital, Fudan University, Shanghai, China; ^4^Institute of Acupuncture and Moxibustion, Fudan University, Shanghai, China; ^5^Institute of Integrative Medicine, Fudan University, Shanghai, China; ^6^Department of Clinical Neuroscience, Karolinska Institutet, Center for Molecular Medicine, Karolinska University Hospital, Stockholm, Sweden

## Abstract

Electroacupuncture has been shown to promote remyelination in a demyelinating model of multiple sclerosis (MS) through enhanced microglial clearance of degraded myelin debris. However, the mechanisms involved in this process are yet to be clearly elucidated. It has been revealed that TAM receptor tyrosine kinases (Tyro3, Axl, and MerTK) play pivotal roles in regulating multiple features of microglia, including the phagocytic function and myelin clearance. Therefore, the aim of this study is to further confirm whether electroacupuncture improves functional recovery in this model and to characterise the involvement of the TAM receptor during this process. In addition to naive control mice, a cuprizone-induced demyelinating model was established, and long-term electroacupuncture treatment was administrated. To evaluate the efficiency of functional recovery following demyelination, we performed beam-walking test and rotarod performance test; to objectify the degree of remyelination, we performed transmission electron microscopy and protein quantification of mature oligodendrocyte markers. Oil Red O staining was used to evaluate the deposit of myelin debris. We confirmed that, in cuprizone-treated mice, electroacupuncture significantly ameliorates motor-coordinative dysfunction and counteracts demyelinating processes, with less deposit of myelin debris accumulating in the corpus callosum. Surprisingly, mRNA expression of TAM receptors was significantly upregulated after electroacupuncture treatment, and we further confirmed an increased protein expression of Axl and MerTK after electroacupuncture treatment, indicating their involvement during electroacupuncture treatment. Finally, LDC1267, a selective TAM kinase inhibitor, abolished the therapeutic effect of electroacupuncture on motor-coordinative dysfunction. Overall, our data demonstrate that electroacupuncture could mitigate the progression of demyelination by enhancing the TAM receptor expression to facilitate the clearance of myelin debris. Our results also suggest that electroacupuncture may be a potential curative treatment for MS patients.

## 1. Introduction

Multiple sclerosis (MS) is a progressive disease characterised by inflammation-induced demyelination of the central nervous system (CNS); currently, MS affects >2.5 million people worldwide [1]. Inflammation, demyelination, loss of oligodendrocytes, and subsequent axonal injury are the common pathological elements of MS [2]. A number of immunosuppressive and immunomodulatory agents can be applied to reduce the frequency of relapse and decrease the rate of damage during MS treatment; however, little attention has been given to myelin repair, and more well-defined therapeutic strategies are therefore required.

Acupuncture, a therapeutic intervention that originated from ancient China [3], has been used as a clinical treatment for various CNS diseases including stroke, Alzheimer's disease, Parkinson's disease, and depression [4–9]. Electroacupuncture differs from standard acupuncture in that a mild electric current passes through pairs of needles during treatment. Studies have shown that the therapeutic effects of electroacupuncture on CNS disease are related to its neuroprotective effect and the prevention of secondary injury [10–12]. In MS treatment, a number of clinical reports have shown that electroacupuncture can significantly improve symptoms such as limb weakness, pain, and numbness in patients [13, 14]; furthermore, electroacupuncture alleviates neuroinflammation and neurological impairment in the classical MS animal model experimental autoimmune encephalomyelitis [11]. Previously, our group found that electroacupuncture treatment from the disease peak improves remyelination in a cuprizone-induced demyelinating model [15]. However, the mechanism that underlies the beneficial effect of electroacupuncture remains unclear, and more methods are needed to further evaluate and confirm the therapeutic effect of electroacupuncture, especially transmission electron microscopy, which is regarded as the gold-standard technique to assess myelination. In addition, we are also curious whether early electroacupuncture treatment could mitigate an ongoing demyelinating process. Our previous bulk tissue RNA-seq data revealed a potential involvement of the TAM receptor family after electroacupuncture treatment. Therefore, we identified the TAM receptor family as a target for further investigation.

The TAM receptors comprise a family of three receptor tyrosine kinases (RTKs): Tyro3, Axl, and MerTK. These receptors, upon ligand binding, promote phagocytosis and immunoregulation. In the CNS, Axl and MerTK are mainly expressed in microglia, and deletion of their associated genes aggravates inflammation and autoimmune diseases [16, 17]. Studies have shown that microglial phagocytosis can clear myelin debris, promote the differentiation of oligodendrocyte progenitor cells (OPCs) to form mature oligodendrocytes, and promote the generation of new myelin sheaths [18, 19]. Furthermore, Axl and MerTK have been shown to participate in clearance of damaged necrotic cells by microglia cells [17, 20, 21]. However, whether the TAM receptor family mediates the beneficial effects of electroacupuncture in the settings of demyelination and remyelination is not known. Therefore, the aims of the present study were as follows: (1) to investigate whether early administration of electroacupuncture could mitigate the ongoing demyelinating process and further confirm its therapeutic effect during remyelination and (2) to elucidate the potential mechanism of electroacupuncture in relation to phagocytic receptors, Axl and MerTK. Given the predictable time course of demyelination/remyelination, the cuprizone model, a toxic demyelination model widely adopted to investigate myelin repair [22], was used here. To our knowledge, this is the first study to investigate the regulatory role of electroacupuncture in TAM receptors.

## 2. Materials and Methods

### 2.1. Cuprizone-Induced Demyelination Models and Experimental Design

**Figure 1:**
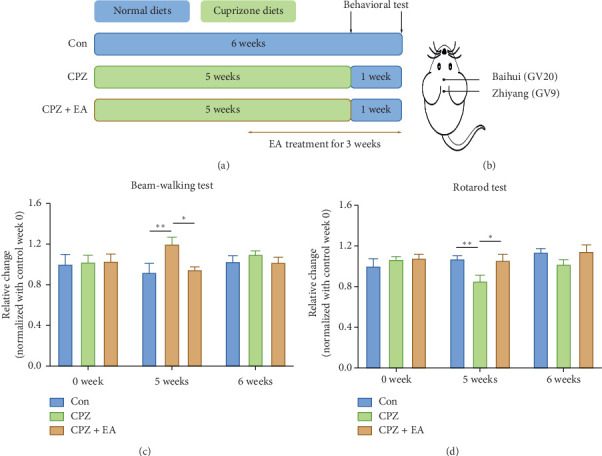
EA ameliorates motor-coordinative dysfunction associated to CPZ administration. (a) The scheme of experiment: CPZ administration is withdrawn after 5 weeks (demyelination phase) followed by 1 week of the remyelination phase. Mice received 3 weeks of EA treatment since the 1st day at week 4 when they started exhibiting severe defects in neurological behaviours. Behavioral test is performed at the end of weeks 5 and 6. (b) The location of Baihui (GV20) and Zhiyang (GV9) acupoints. (c and d) EA treatment significantly reduces the time spent in beam-walking test at week 5. EA treatment significantly increases the time remaining on the cylinder at week 5. All data are represented as means ± SEM (*n* = 6–8 for each group; ^*∗*^*p* < 0.05, ^*∗∗*^*p* < 0.01).

The cuprizone model was produced using 6-week-old C57BL/6 male mice (Experimental Animal Center, Chinese Academy of Sciences, Shanghai, China) following a 1-week adaptation in our animal facility. Cuprizone powder (Sigma-Aldrich, St. Louis, MO, USA) was mixed with standard rodent chow at a ratio of 0.3% and fed to mice for 5 weeks. After 5 weeks, we withdrew cuprizone chow and replaced it with standard chow for 1 week to allow for spontaneous and incomplete remyelination (Figure 1(a)). All experimental protocols were conducted in accordance with the National Institute of Health Guide for the Care and the Animal Research Welfare and approved by the council of the School of Basic Medical Science, Fudan University. We made all endeavours to minimise the sacrifice of experimental animals and alleviate their suffering.

### 2.2. Electroacupuncture and LDC1267 Treatment

Electroacupuncture treatment began from the first day of week 4 and continued for 3 weeks. Mice were given electroacupuncture for 30 min every other day (Figure 1(a)), and the electric current was 4 mA. Mice were subjected to an adaptive process to avoid unnecessary sacrifice. Two acupoints in the governor vessel were adopted, namely, Baihui (GV20) and Zhiyang (GV9) (Figure 1(b)). Stainless steel needles (Suzhou Medical Supplies, Suzhou, P.R. of China) were inserted 5 mm into the two acupoints of mice subcutaneously and obliquely, avoiding the skull and vertebral canal. The pair of needles was then connected with the output terminal of the electroacupuncture apparatus (HANS Acupoint Nerve Stimulator, LH202H, Beijing, P.R. of China), which produced alternating dense/sparse frequencies of 2/15 Hz. The mice were immobilised on a specially designed apparatus made in our lab for electroacupuncture treatment. In our preliminary study, we included a sham-electroacupuncture group that was subjected to the same acupuncture treatment but without electrical stimulation. In some experiments, LDC1267 (20 mg/kg via intraperitoneal injection; Selleckchem, USA), a highly selective TAM kinase inhibitor, was administered 30 min prior to electroacupuncture in order to evaluate the role of TAM receptors in the effects of electroacupuncture. The control mice received the same volume (200 *μ*l) of the delivery vehicle (2% DMSO + 40% PEG400 + 2% Tween80 + ddH_2_O) without LDC1267.

### 2.3. Beam-Walking Test

A beam-walking test was used to evaluate the locomotor coordination of the mice. In brief, a narrow wooden beam was placed 60 cm above a platform, which was paved with thick cotton cushions. The day before the test, we trained the mice to reach a padded “safety box” via a 1 m long/1 cm wide balance beam, i.e., a relatively safe pathway for the mice to traverse to reach the safety box. The test began at the same time on the next day, and the mice's traversing times were recorded. In order to reduce error, the time that the mice spent on both ends (10 cm portions) of the balance beam was neglected; thus, the time taken for the mice to traverse the middle 80 cm of the beam was recorded. Each mouse was tested twice consecutively, and the mean time of travel was calculated. The test was conducted at week 0 (the day before modelling) first, and the obtained value was also used to confirm that no significant differences in locomotor coordination function existed among the groups before modelling began.

### 2.4. Rotarod Performance Test

A rotarod test was used to evaluate the motor coordination and balance of the mice. The test involved an accelerating rod and a mouse placed on a rotating cylinder. The day before the test, each mouse was trained on the cylinder, which was accelerated gradually from 4 rpm to 40 rpm (0.1 rpm per second) and allowed to run for 300 s. Training improved the animals' skill and avoided fortuitous falling. If the mice fell or remained on the cylinder for two cycles, the test was stopped. The time for which each animal was able to maintain its balance on the rotating cylinder was recorded. All mice were tested on the rotarod twice. The average latency was calculated from two consecutive measurements. The test was conducted at week 0 (the day before modelling) first, and the base value of motor coordination was also compared among groups to confirm that there was no significant difference among groups prior to modelling.

### 2.5. Western Blotting

For western blotting, corpus callosum tissue of mice was quickly resected on ice and ultrasonically homogenised in RIPA buffer supplemented with 1 mM PMSF and protease inhibitor cocktail. Equal amounts of protein samples (∼20 *μ*g) from each group were loaded in SDS-PAGE gels and transferred to polyvinylidene fluoride (PVDF) membranes. Membranes were incubated with the following primary antibodies: antimyelin basic protein (MBP) (1 : 400, Merck Millipore), anti-MerTK (1 : 1000, R&D Systems), anti-Axl (1 : 1000, R&D Systems), anti-Tyro3 (1 : 1000, R&D Systems), anti-Gas6 (1 : 1000, R&D Systems), and anti-*β*-actin (1 : 10000, ProteinTech). Protein expression was detected using an enhanced chemiluminescence detection system (Tanon) via incubation with peroxidase-conjugated secondary antibodies. Images were obtained using an ImageQuant LAS4000 mini image analyser (GE Healthcare).

### 2.6. Quantitative RT-PCR (qRT-PCR) Analysis

Total RNA was isolated using Trizol reagent (Invitrogen). cDNA was synthesised using a PrimeScript RT Reagent Kit with gDNA Eraser (Takara) following the manufacturer's recommendations. The relative abundance of target mRNAs was quantified using SYBR Green qRT-PCR detection (LightCycler 96 real-time PCR detection system, Roche). The housekeeping gene GAPDH was used as an internal reference for the purposes of standardisation during the analysis. The primer sequences of target mRNAs are listed in Table 1. Relative quantification was performed by determination of the n-fold differential expression with the 2^−ΔΔCT^ method, and the results are expressed as the relative fold change in comparison to GAPDH.

### 2.7. Immunofluorescence Staining

Mice were anaesthetised with an intraperitoneal injection of pentobarbital sodium and perfused with cold saline solution followed by 4% formaldehyde. Their brains were dissected and then immersed in 4% formaldehyde for fixation followed by dehydration with sucrose solution. The coronal brain sections used for histological analysis were derived mostly from the caudal callosum, beginning at approximately −0.5 mm from bregma. The brain sections (25 *μ*m) were first washed three times with PBS and then blocked with 4% goat serum in 0.3% Triton X-100 for 1 h at room temperature. Subsequently, they were incubated overnight at 4°C with the anti-MBP (1 : 400, Merck Millipore), anti-Iba1 (1 : 400, Wako, Japan), anti-MerTK (1 : 100, R&D Systems), or anti-Axl (1 : 100, R&D Systems) primary antibodies. Samples were then stained with Alexa Fluor 488 or 594 secondary antibodies (1 : 1000, Invitrogen) at room temperature for 2 h before rinsing with PBS. Sections were mounted with fluorescence decay resistant medium (with DAPI) before they were covered with a cover slip. Images were captured using a confocal microscope (Olympus FluoView FV1000).

### 2.8. Oil Red O Staining

The accumulation of myelin debris is detrimental to the remyelination of axons [23]; therefore, timely removal of myelin debris during demyelination is critical for subsequent remyelination [19]. To determine if there was less myelin debris in electroacupuncture-treated mice, Oil Red O staining was used to detect the presence of lipid droplets around lesions. First, the brain slices were incubated with 60% propylene glycol for 2 min after rinsing and then stained with Oil Red O working solution at 60°C for 10 min before being rinsed again. Nuclei were stained with hematoxylin.

### 2.9. Transmission Electron Microscopy (TEM)

To further evaluate demyelination injury, the mice were anaesthetised at the end of the study, and 1 mm^3^ of corpus callosum tissue was dissected on ice immediately after death. These samples were immersed in 2.5% glutaraldehyde TEM fixation solution (Servicebio) and 1% osmic acid in 0.1 M PBS (pH 7.4). After fixation and dehydration with ethanol, the tissue was embedded using acetone and embedding medium (Servicebio). Ultrathin sections were prepared using an ultramicrotome (Leica UC7). The ultrastructure of myelinated axons was imaged using the FEI Tecnai G2 20 TWIN electron microscope.

### 2.10. Statistical Analysis

All data are represented as mean ± SEM. Quantitative data were analysed and graphed using GraphPad Prism 5. Intergroup statistical data were analysed using two-way ANOVA followed by Tukey posttest. Values of *p* < 0.05 were considered as statistically significant.

## 3. Results

### 3.1. Electroacupuncture Ameliorates Motor-Coordinative Dysfunction Associated with Cuprizone Administration

Cuprizone administration causes oligodendrocyte cell death, which leads to extensive demyelination in the CNS, with the corpus callosum being the region predominantly affected, and cuprizone-fed mice develop motor-coordinative dysfunction [24–26]. To test the effect of electroacupuncture on motor coordination in the cuprizone model, the beam-walking and rotarod tests were performed before and after electroacupuncture treatment at different time points (Figure 1(a)). There were no differences in the beam-walking and rotarod performance of mice among the experimental groups at week 0. In contrast, at the end of week 5, which is also the end point of the demyelinating phase, cuprizone model mice took significantly longer time to traverse the beam, and their performance was accompanied by more hesitation (Figure 1(c)). Consistent with the beam-walking results, the latency of the cuprizone-fed mice on the accelerated rod was significantly shorter than that of the naive control group at week 5 (Figure 1(d)). These results, in accordance with many previous studies, suggest that cuprizone-fed mice develop motor-coordinative dysfunction [15, 27]. After 2 weeks of electroacupuncture, the time for mice to traverse the balance beam had significantly reduced (Figure 1(c)), and the latency of these mice on the rotarod had also increased (Figure 1(d)). These results indicate that electroacupuncture treatment is effective for containing motor coordinative deficits during active demyelinating process. After maintenance of cuprizone diet for 5 weeks, mice were given normal diet for 1 week, which enables them to spontaneous remyelination, a process also evident in elapsing-remitting MS patients following each disease attack. Surprisingly, during our experiments, the cuprizone-treated mice exhibited a fast recovery at week 6 as there were no significant differences in beam-walking or rotarod performance among the groups, but a similar trend was observed at week 5.

### 3.2. Electroacupuncture Treatment Mitigates the Ongoing Process of Demyelination

**Figure 2:**
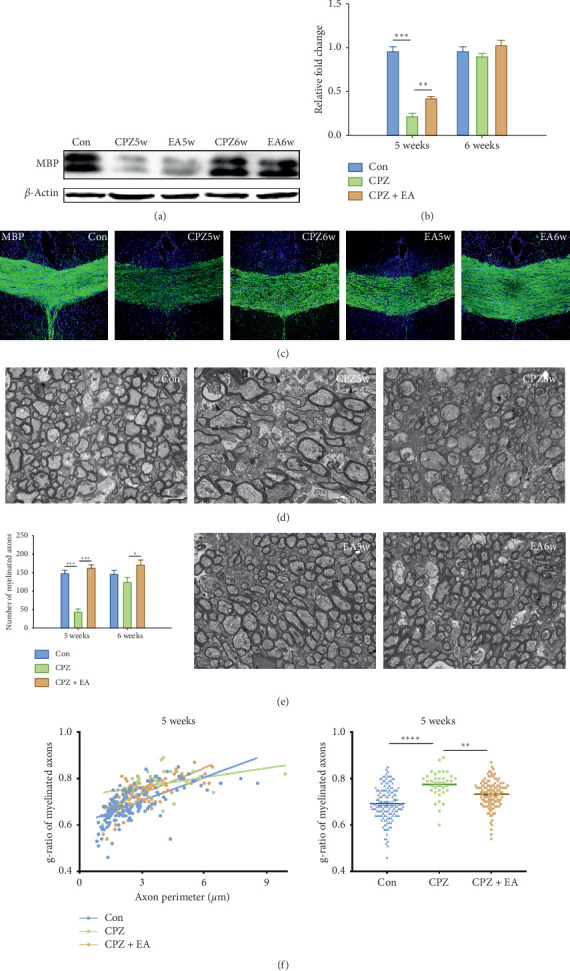
EA treatment reduces CPZ-induced demyelination. (a) The protein expression of MBP in each group at weeks 5 and 6. EA treatment significantly increases the expression of myelin-related protein. (b) Bar graph shows the quantification of the MBP expression by western blot (*n* = 5 for each group). Consistently, (c) immunostaining of MBP shows the effect of promoting remyelination by EA treatment (scale bar = 50 *μ*m). (d and e) Ultrastructure of axon/myelin by TEM. EA treatment significantly increases the percentage of the myelinated axon per image field (scale bar = 2 *μ*m) and decreases the number of swollen axons (black arrows). (f) Scatter plot of individual g-ratio against axonal perimeter (left panel) and the integrated g-ratio values among groups (right panel). All data are represented as means ± SEM (^*∗*^*p* < 0.05, ^*∗∗*^*p* < 0.01, and ^*∗∗∗*^*p* < 0.001).

We have previously shown that electroacupuncture treatment promotes remyelination during the remyelination phase in the cuprizone model [15], and here, we further investigate whether electroacupuncture has an impact on the ongoing demyelinating process in the cuprizone model. MBP, a marker protein of mature oligodendrocytes and myelin sheath, was evaluated at the protein level by western blotting and immunohistochemistry. Results from western blotting showed that the MBP expression was significantly decreased after 5 weeks of cuprizone feeding; however, electroacupuncture significantly attenuated the loss of MBP at the end of week 5 (Figures 2(a) and 2(b); two-way ANOVA, Tukey's post hoc test of MBP/actin ratios in the following (*n* = 5 per group): control vs. cuprizone 5 weeks, *p* < 0.001; cuprizone 5 weeks vs. cuprizone + electroacupuncture 5 weeks, *p* < 0.01). However, the difference was not significant among the three groups at the end of week 6, indicating a rapid recovery in the batch of the cuprizone model we induced, which is also consistent with the behavioral results (Figures 2(a) and 2(b); MBP/actin ratio (*n* = 5 per group): control = 0.961 ± 0.115, cuprizone 5 weeks = 0.223 ± 0.065, cuprizone + electroacupuncture 5 weeks = 0.428 ± 0.040, cuprizone 6 weeks = 0.905 ± 0.068, and cuprizone + electroacupuncture 6 weeks = 1.035 ± 0.109). The results from MBP immunofluorescent staining in the corpus callosum were consistent with the western blotting results. Specifically, myelin loss was evident in the midline corpus callosum after 5 weeks of cuprizone feeding, but electroacupuncture increased the staining for MBP in demyelinated areas of the corpus callosum (Figure 2(c)). The anti-demyelinating potential of electroacupuncture was further evaluated at the ultrastructural level by TEM. Cuprizone administration led to a significant reduction in the percentage of myelinated axons per image field at the end of week 5 (Figures 2(d) and 2(e)) and was associated with more oedema and swollen axons. In contrast, after electroacupuncture treatment, there was a significant increase in the percentage of myelinated axons at weeks 5 and 6 (Figures 2(d) and 2(e)). In addition, the cuprizone-treated mice showed a higher g-ratio (the ratio between the inner and the outer diameter of the myelin sheath) compared with control mice, while electroacupuncture-treated mice exhibited decreased g-ratio of myelinated axons (Figure 2(f)). Taken together, we demonstrated here that, in addition to a previously reported pro-remyelinating effect, electroacupuncture treatment from early time point could also mitigate the process of ongoing demyelination.

### 3.3. Electroacupuncture Promotes the Clearance of Degraded Myelin Debris by Recruiting More Microglial Cells into the Corpus Callosum

**Figure 3:**
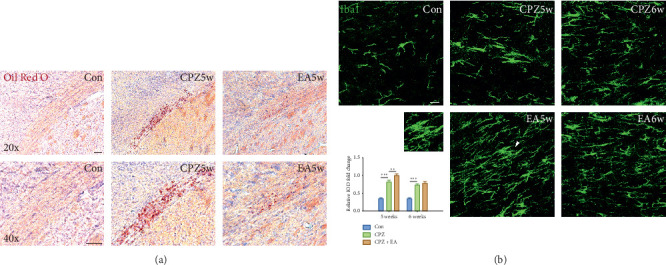
EA promotes the clearance of degraded myelin debris. (a) Oil Red O staining shows that the lipid-associated deposit of degraded myelin debris is eliminated after 2 weeks of EA treatment. (b) Microglia assembling into the corpus callosum at weeks 5 and 6 is detected by Iba1 (green), and representative microglia displaying the amoeboid and phagocytic shape after EA treatment with higher magnification (scale bar = 20 *μ*m). All data are represented as means ± SEM (for statistical graph: *n* = 5 for each group; ^*∗*^*p* < 0.05, ^*∗∗*^*p* < 0.01, and ^*∗∗∗*^*p* < 0.001).

Accumulation of myelin debris is a main feature of demyelination, and since myelin debris inhibits the differentiation of oligodendrocyte precursors, efficient clearance of myelin debris is determinant for remyelination [19, 23]. Oil Red O staining was used to detect the presence of lipid droplets around lesions as well as the accumulation of myelin debris [17]. Therefore, we also introduced Oil Red O staining here to evaluate the accumulation of myelin debris. In cuprizone-fed mice, the robust presence of Oil Red O-positive lipid droplets in the corpus callosum suggested the presence of myelin debris. As expected, electroacupuncture significantly reduced the accumulation of lipid droplets at the end of week 5 (Figure 3(a)). Recent evidence suggests that microglia play a neuroprotective role by removing myelin debris and recruiting OPCs [28–31]. Accumulation of microglia was investigated by immunolabelling with Iba-1. According to immunolabelling with Iba-1, microglia in naive control mice displayed a ramified morphology with small cell soma and thinner processes, whereas in the cuprizone-fed group as well as in the electroacupuncture group, Iba-1 immunoreactivity significantly increased, and microglial morphology was characterised by enlarged cell soma and thickened cell processes (Figure 3(b)). Interestingly, electroacupuncture apparently enhanced the recruitment of microglia as evidenced by more Iba-1-positive cells in the corpus callosum at the end of week 5 (Figure 3(b)). These data suggest that recruitment of microglial cells was promoted by electroacupuncture, contributing to the clearance of myelin debris.

### 3.4. Electroacupuncture Increases the Expression of Phagocytosis-Related Receptors MerTK and Axl

**Figure 4:**
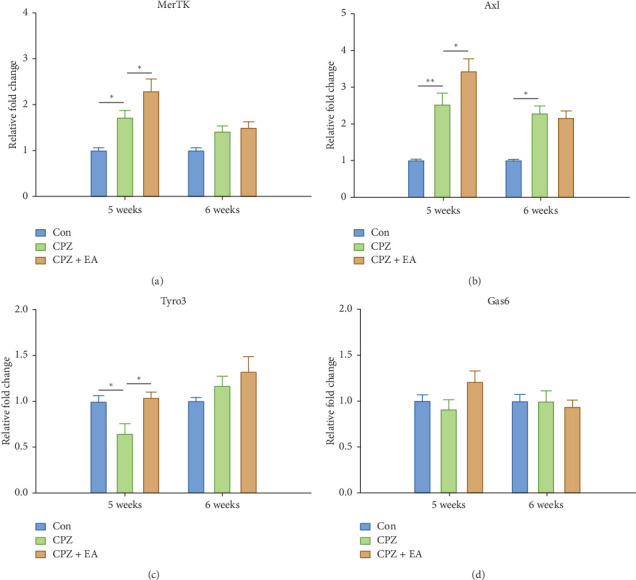
EA increases the mRNA expression of phagocytosis-related receptors MerTK and Axl in the corpus callosum. (a–d) mRNA level of genes of the TAM receptor family in each group at weeks 5 and 6 (*n* = 4–8 for each group; all data are represented as means ± SEM ^*∗*^*p* < 0.05; ^*∗∗*^*p* < 0.01).

It was previously reported that the TAM receptor tyrosine kinases MerTK and Axl are important functional regulators of myelin phagocytosis [32–34], and MS patients have defect in myelin phagocytosis by myeloid cells due to decreased expression of MerTK. To determine the effect of electroacupuncture on the phagocytosis-associated receptors, we first analysed the expression of genes encoding the TAM receptors MerTK, Axl, and Tyro3, and the ligand Gas6, using qRT-PCR. As shown in Figures 4(a) and 4(b), the mRNA expression of *MerTK* and *Axl* increased 1.712-fold (*n* = 7, *p* < 0.05) and 2.524-fold (*n* = 8, *p* < 0.01), respectively, at week 5 in cuprizone-fed mice compared with the naive control group. In addition, the upregulated expression of *Axl* in cuprizone-fed mice was also evident at week 6 (*n* = 8, *p* < 0.05 vs. control). In contrast to *MerTK* and *Axl*, the expression of *Tyro3* was significantly lower in mice after 5 weeks of cuprizone challenge (*n* = 6, *p* < 0.05 vs. control; Figure 4(c)). However, after 6 weeks, expression of *Tyro3* and *MerTK* in the cuprizone model was no longer significantly different than that in the naive control group. Furthermore, the mRNA levels of *MerTK*, *Axl*, and *Tyro3* increased significantly at week 5 in mice receiving electroacupuncture treatment. However, the expression of *Gas6* was not significantly altered at the two time points among these groups (Figure 4(d)).

**Figure 5:**
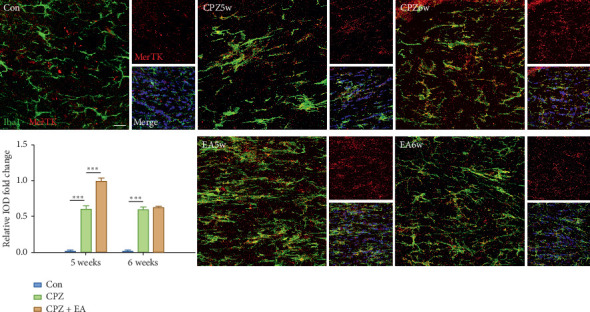
EA upregulates the expression of MerTK in the corpus callosum. Immunofluorescence staining of MerTK (red), Iba1 (green), and merge (scale bar = 20 *μ*m). All data are represented as means ± SEM (for statistical graph: *n* = 4–6 for each group; ^*∗*^*p* < 0.05, ^*∗∗*^*p* < 0.01, and ^*∗∗∗*^*p* < 0.001).

**Figure 6:**
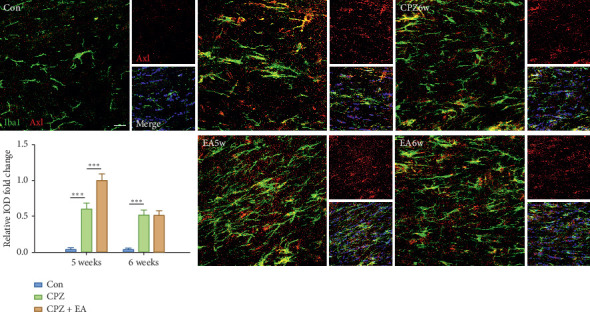
EA upregulates the expression of Axl in the corpus callosum. Immunofluorescence staining of Axl (red), Iba1 (green), and merge (scale bar = 20 *μ*m). All data are represented as means ± SEM (for statistical graph: *n* = 4-5 for each group; ^*∗*^*p* < 0.05, ^*∗∗*^*p* < 0.01, and ^*∗∗∗*^*p* < 0.001).

We next analysed the expression of MerTK and Axl at the protein level by immunohistochemistry, and since microglia are the main phagocytic cells in the CNS, we also double-stained MerTK/Axl with Iba1. Notably, there were very few MerTK-immunoreactive or Axl-immunoreactive cells in the corpus callosum of control mice. However, in cuprizone-induced mice, dense and widespread MerTK-positive and Axl-positive cells were observed. Most MerTK- or Axl-positive cells also expressed Iba-1, suggesting that microglia are a major source of these two TAM receptors (Figures 5 and 6). Electroacupuncture treatment further significantly enhanced the protein expression of MerTK and Axl at the end of week 5. These data confirmed the involvement of phagocytic receptors MerTK and Axl during electroacupuncture treatment in the cuprizone model.

### 3.5. The TAM Kinase Inhibitor LDC1267 Abolishes the Therapeutic Effect of Electroacupuncture on Motor-Coordinative Dysfunction

**Figure 7:**
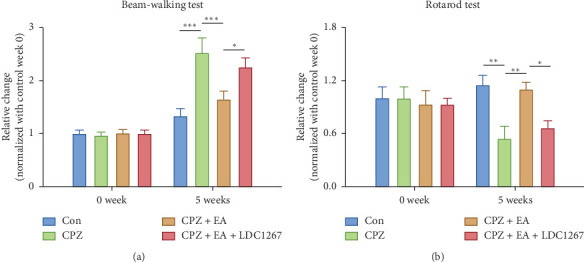
TAM kinase inhibitor LDC1267 abolishes the therapeutic effect of EA on motor dysfunction. EA treatment significantly reduces the time spent in beam-walking test and increases the time remaining on the cylinder at week 5. LDC1267 eliminates the effects of EA treatment. All data are represented as means ± SEM (*n* = 6–8 for each group; ^*∗*^*p* < 0.05, ^*∗∗*^*p* < 0.01, and ^*∗∗∗*^*p* < 0.001).

To further verify the role of TAM receptors in the therapeutic effects of electroacupuncture, the highly selective TAM kinase inhibitor, LDC1267, was administrated prior to electroacupuncture treatment. We found that daily administration of LDC1267 (20 mg/kg body weight) markedly reduced the improvement in motor-coordinative function previously observed following electroacupuncture treatment (Figure 7). These suggest that the functional improvement by electroacupuncture treatment following demyelination also requires the involvement of TAM receptors.

## 4. Discussion

Halting the process of demyelination and promoting remyelination as well as axonal regeneration are the pathological basis for the improvement of neuronal function and clinical symptoms for patients with demyelinating diseases, including MS. Thus, finding a safe and effective treatment that can be used clinically, with evidence elucidating its underlying mechanism, represents a currently significant medical need. Here, we have shown that early administration of electroacupuncture ameliorates motor-coordinative dysfunction and reduces demyelination associated with the administration of cuprizone in mice. Similarly, the severity of clinical symptoms of MS patients, which are evaluated by a standardised neurological examination (Expanded Disability Status Scale) that focuses on the frequent symptoms of MS and is used in clinical trials of MS for the assessment of disability [35], was also improved in MS patients that received electroacupuncture [13, 36–39]. In addition to the beneficial effect on neurological functions, acupuncture also showed therapeutic benefits for a proportion of patients with fatigue, gait impairment, pain, and bladder dysfunction [13, 39, 40]. In line with our study, the positive effects of acupuncture in the MS-like model have also been demonstrated in other animal models of MS: in the classic experimental autoimmune encephalomyelitis (EAE) model, electroacupuncture could modulate T cell response and modulate Th1/Th2/Th17/Treg subsets by increasing ACTH secretion in the thalamus; in an ethidium bromide-induced demyelinating model, electroacupuncture intervention decreased disease severity and increased the production and differentiation of OPCs, facilitating remyelination [41, 42].

In the current study, we used the cuprizone-induced demyelinating model, in which apoptosis of oligodendrocytes and loss of the myelin sheath occur following toxic exposure, and subsequent gliosis is evident at 3-4 weeks after cuprizone feeding. Unlike the EAE model, which is characterised by the massive inflammatory response with CNS cytokine burst, neuroinflammation is very mild in the cuprizone model and is regarded secondary to CNS damage and could be a response to promote repair following myelin damage [43, 44]. The motor dysfunction of cuprizone-treated mice is usually subtle to observe as they appear normal, without obvious muscle weakness, paralysis, or any other visible physical signs as seen in the EAE model. However, by performing behavioral test such as beam-walking test, pole test, and rotarod test, it was demonstrated by others and our group that cuprizone-treated mice show affected locomotor-coordinative function [15, 25, 27, 45, 46]. In general, most studies [24, 47, 48] used 6- to 9-week-old mice maintaining a diet containing 0.2–0.3% cuprizone for 5-6 weeks to induce CNS demyelination. The outcome effect of cuprizone-induced intoxication could vary depending on many factors, such as mouse strain, age, gender, and even the local environment in the animal facility [49, 50]. Here, we used 0.3% cuprizone supplement in our animal facility since our previous pilot experiments reported insufficient demyelination or weight loss/behavioral deficits using 0.2%. Of note, compared to previous studies [47, 50], we found a faster recovery after the withdrawal of cuprizone for 1 week, indicating a potential batch discrepancy when inducing the cuprizone model.

In the present study, lipid-associated myelin debris (shown by Oil Red O staining) decreased following electroacupuncture, which corresponds with the accumulation of microglia. Consistent with these results, our previous research showed that electroacupuncture was an efficient therapeutic intervention to modulate microglial function [15]. Myelin debris are pathological products that accumulate in lesion areas during cuprizone treatment and the process of demyelination in MS patients; moreover, they are potent obstacles for remyelination as well as for axon regeneration [23]. Microglia are vital for maintaining homeostasis in the normal CNS due to their constant phagocytic clearance of cell debris; this role is amplified beyond simple debris clearance during CNS injury, in which microglia also play a crucial role in the reorganisation of neuronal circuits and triggering repair [18]. Indeed, several studies have reported the beneficial effects of microglia, which include axonal regeneration, promotion of remyelination, clearance of inhibitory myelin debris, and the release of neurotrophic factors [51]. Furthermore, loss-of-function studies have demonstrated that efficient remyelination cannot take place if clearance of myelin debris by microglia is dysfunctional [17, 19]. Moreover, our results are in agreement with studies from other animal models; for example, electroacupuncture had a positive impact on a rat spinal cord injury model, which may have been related to an increase in the polarisation of M2 microglia [52].

The TAM receptors, Tyro3, Axl, and MerTK, play an essential role in regulating homeostasis in adult tissues and organ systems. For example, TAMs and their ligands Gas6 and protein S are imperative for the efficient phagocytosis of apoptotic cells and membranes [53–57]. In addition, these receptors can recognise phosphatidylserine as an “eat me” signal through their ligands for engulfment by phagocytes [58]. They are highly expressed in the CNS; Axl and MerTK are mostly expressed in microglia and astrocytes, whereas Tyro3 is relatively abundant in neurons. TAM receptors, especially MerTK, are proposed as very important regulators of myelin phagocytosis [33]. In the present study, electroacupuncture significantly increased the expression of the phagocytosis-related receptors Axl and MerTK in cuprizone-model mice. We found that although Axl and MerTK are enriched in microglia, they are not exclusively expressed in microglia. This is also in favor of the recent proposed notions that although microglia were regarded as the main scavenger in the CNS, reactive astrocytes (also express TAM receptors) could transform into a phagocytic phenotype and are also responsible for myelin clearance [59, 60].

Taken together, our study showed that the important regulators of myelin clearance, MerTK and Axl, are significantly upregulated after electroacupuncture treatment, and it could be an underlying mechanism for the effect of electroacupuncture in combating demyelinating diseases. Nevertheless, there could still be many other factors mediating the therapeutic effect of electroacupuncture, and it requires additional research to be fully elucidated.

## Figures and Tables

**Table 1:** Primers used for RT-PCR analysis.

Genes	Species	Sequence (5′-greater than 3′)
*MerTK*	Mouse	FW: CAGGGCCTTTACCAGGGAGA
		RE: TGTGTGCTGGATGTGATCTTC
*Axl*	Mouse	FW: ATGGCCGACATTGCCAGTG
		RE: CGGTAGTAATCCCCGTTGTAGA
*Tyro3*	Mouse	FW: GCCTCCAAATTGCCCGTCA
		RE: CCAGCACTGGTACATGAGATCA
*Gas6*	Mouse	FW: GGGCCTAAAACTATCCCCAGA
		RE: GGTACAAGGACTTCACGCTCT
*GADPH*	Mouse	FW: GGTTGTCTCCTGCGACTTCA
		RE: TGGTCCAGGGTTTCTTACTCC

## Data Availability

The data used to support the findings of this study are available from the corresponding author (Jun Wang) upon request.
